# Supplementary material for: Mast cells promote pathology and susceptibility in tuberculosis
Source: eLife. 2026 Jan 28;13:RP102634. doi: 10.7554/eLife.102634 (PMC12851577; doi:10.7554/eLife.102634)
Supplement: MDAR checklist [file elife-102634-mdarchecklist1.docx]

**Materials Design Analysis Reporting (MDAR)**

**Checklist for Authors**

The [MDAR framework](https://osf.io/xfpn4/) establishes a minimum set of requirements in transparent reporting mainly applicable to studies in the life sciences.

*eLife* asks authors to **provide detailed information within their article** to facilitate the interpretation and replication of their work. Authors can also upload supporting materials to comply with relevant reporting guidelines for health-related research (see [EQUATOR Network](http://www.equator-network.org/%20)), life science research (see the [BioSharing Information Resource](http://biosharing.org/)), or animal research (see the [ARRIVE Guidelines](http://www.plosbiology.org/article/info:doi/10.1371/journal.pbio.1000412) and the [STRANGE Framework](https://doi.org/10.1038/d41586-020-01751-5); for details, see *eLife*’s [Journal Policies](https://reviewer.elifesciences.org/author-guide/journal-policies)). Where applicable, authors should refer to any relevant reporting standards materials in this form.

For all that apply, please note **where in the article** the information is provided. Please note that we also collect information about data availability and ethics in the submission form.

**Materials:**

| **Newly created materials** | **Indicate where provided: section/figure legend** | **N/A** |
| --- | --- | --- |
| The manuscript includes a dedicated "materials availability statement" providing transparent disclosure about availability of newly created materials including details on how materials can be accessed and describing any restrictions on access. |  | N/A |
|  |  |  |
| **Antibodies** | **Indicate where provided: section/figure legend** | **N/A** |
| For commercial reagents, provide supplier name, catalogue number and [RRID](https://scicrunch.org/resources), if available.  CD11b-APC (clone M1/70, BD Biosciences), CD11c-PE-Cy7 (clone HL3, BD Biosciences), GR-1-PerCP-Cy5.5 (clone RB6-8C5, BD Pharmingen), and MHC class II-FITC (clone M5/114.15.2, Tonbo Biosciences), CD117 (cKit)-Super Bright 780 (clone 2BB, eBioscience), and FcεR1-PE (clone MAR-1, eBioscience), CD3-AF700 (clone 500A2, BD Biosciences), CD4-Pacific Blue (clone RM4.5, BD Biosciences), CD44-PE-Cy7 (clone 1M7, Tonbo Biosciences), and CD8-APC-Cy7 (clone 53-6.7, BD Biosciences, IFNγ-APC (clone XMG1.2, Tonbo Biosciences), TNF-α-FITC (clone MP6-XT22, BD Pharmingen), APC rat IgG1κ and FITC rat IgG1α, BD Pharmingen.  APC-conjugated rat anti-mouse Ly6G (clone 1A8, BioLegend, RRID:AB_2227348), Goat anti-human mast cell chymase (LifeSpan Biosciences, LS-B4134, RRID: AB_10718418,) and rabbit anti-human tryptase (Cell Signaling Technology, 195235), Alexa Fluor 568 donkey anti-goat IgG (Thermo Fisher Scientific, A-11057, RRID: AB_2534104), and Alexa Fluor 488 donkey anti-rabbit IgG (Jackson ImmunoResearch Laboratories, 711-546-152, RRID: AB_2340619). | Location: Materials and Methods-Generation of single-cell suspensions from tissues and flow cytometry staining  and Morphometric analysis of lung Histopathology and neutrophil infiltration |  |
|  |  |  |
| **DNA and RNA sequences** | **Indicate where provided: section/figure legend** | **N/A** |
| Short novel DNA or RNA including primers, probes: Sequences should be included or deposited in a public repository. |  | N/A |
|  |  |  |
| **Cell materials** | **Indicate where provided: section/figure legend** | **N/A** |
| Cell lines: Provide species information, strain. Provide accession number in repository OR supplier name, catalog number, clone number, OR RRID. |  | N/A |
| Primary cultures: Provide species, strain, sex of origin, genetic modification status.  Bone marrow-derived mast cells (BMMCs) were generated from WT C57BL/6 mice for which the details of differentiation and phenotyping are provided. | Location: Materials and Methods- Invitro culture and intratracheal delivery of MCs |  |
|  |  |  |
| **Experimental animals** | **Indicate where provided: section/figure legend** | **N/A** |
| Laboratory animals or Model organisms: Provide species, strain, sex, age, genetic modification status. Provide accession number in repository OR supplier name, catalog number, clone number, OR RRID.  Species: Mus musculus; Strain: C57BL/6 (#000664) and B6.Cg-*Kit^W-sh^*/HNihrJaeBsmJ (#030764); Sex: both males and females; Age: 6 weeks old; Genetic modification: The spontaneous *Kit^W-sh^* (or”sash”) mutation in C57BL/6 background, affects melanoblast and mast cell survival; Source: Jackson Laboratories, RRID: IMSR_JAX:030764  Species: Macaca mulatta; Sex: both males and females; Age: 4-16 years; Modification: None; Source: Tulane National Primate Research Center (TNPRC) Institutional Animal Care and Use Committee | Location: Materials and Methods-Study subjects and animal studies and aerosol infection |  |
| Animal observed in or captured from the field: Provide species, sex, and age where possible. |  | N/A |
|  |  |  |
| **Plants and microbes** | **Indicate where provided: section/figure legend** | **N/A** |
| Plants: provide species and strain, ecotype and cultivar where relevant, unique accession number if available, and source (including location for collected wild specimens). |  | N/A |
| Microbes: provide species and strain, unique accession number if available, and source.  *Mycobacterium tuberculosis*  HN878 strain; Source: BEI resources.  CDC1551 strain; Source: BEI resources | Location: Materials and Methods - Aerosol infection |  |
|  |  |  |
| **Human research participants** | **Indicate where provided: section/figure legend) or state if these demographics were not collected** | **N/A** |
| If collected and within the bounds of privacy constraints report on age, sex, gender and ethnicity for all study participants.  Human lung samples obtained from individuals with PTB and LTBI; age and sex included where allowed. | Location: Materials and Methods - Study subjects and animal studies |  |

**Design:**

| **Study protocol** | **Indicate where provided: section/figure legend** | **N/A** |
| --- | --- | --- |
| If the study protocol has been pre-registered, provide DOI. For clinical trials, provide the trial registration number OR cite DOI. |  | N/A |
|  |  |  |
| **Laboratory protocol** | **Indicate where provided: section/figure legend** | **N/A** |
| Provide DOI OR other citation details if detailed step-by-step protocols are available.  For aerosol *Mtb* HN878 infection- Khader, S. A. et.al. (2007). IL-23 and IL-17 in the establishment of protective pulmonary CD4+ T cell responses after vaccination and during Mycobacterium tuberculosis challenge. *Nat Immunol*, *8*(4), 369-377. <https://doi.org/10.1038/ni1449>  For aerosol *Mtb* CDC1551 infection in NHPs: Esaulova, E. et.al., (2021). The immune landscape in tuberculosis reveals populations linked to disease and latency. *Cell Host Microbe*, *29*(2), 165-178 e168. <https://doi.org/10.1016/j.chom.2020.11.013>  For lung single cell preparations: Gopal, R., Rangel-Moreno, J., Slight, S., Lin, Y., Nawar, H. F., Fallert Junecko, B. A., Reinhart, T. A., Kolls, J., Randall, T. D., Connell, T. D., & Khader, S. A. (2013). Interleukin-17-dependent CXCL13 mediates mucosal vaccine-induced immunity against tuberculosis. *Mucosal Immunol*, *6*(5), 972-984. doi: [10.1038/mi.2012.135](https://doi.org/10.1038/mi.2012.135) | Location: Materials and Methods- Aerosol infection and Generation of single-cell suspensions from tissues and flow cytometry staining |  |
|  |  |  |
| **Experimental study design (statistics details) *** | | |
| **For in vivo studies: State whether and how the following have been done** | **Indicate where provided: section/figure legend. If it could have been done, but was not, write “not done”** | **N/A** |
| Sample size determination – Based on historical data | Location: Figure legends |  |
| Randomisation - Animals were randomly assigned to the groups | Location: Material methods and figure legends |  |
| Blinding - Investigators were blinded for CFU enumeration and histopathology scoring. | Location: Material and Methods-Morphometric analysis of lung Histopathology and neutrophil infiltration |  |
| Inclusion/exclusion criteria - All surviving animals included, no exclusion | Location: Materials and Methods- Study subjects and animal studies |  |
|  |  |  |
| **Sample definition and in-laboratory replication** | **Indicate where provided: section/figure legend** | **N/A** |
| State number of times the experiment was replicated in the laboratory-  All the major findings were reproduced from 1-2 independent experiments | Location: Figure legends |  |
| Define whether data describe technical or biological replicates.  Biological replicates: independent animals per group and independent experiments repeated 1-2 times | Location: Figure legends |  |
|  |  |  |
| **Ethics** | **Indicate where provided: section/submission form** | **N/A** |
| Studies involving human participants: State details of authority granting ethics approval (IRB or equivalent committee(s), provide reference number for approval.  All human lung biopsy samples were obtained from the Tuberculosis Outpatient Clinic and the Department of Pathology at the National Institute of Respiratory Diseases (INER) - IRB approval and protocol numbers B04-15 and B09-23.  The analysis was conducted at Washington University in St. Louis, School of Medicine, and approved by the IRB with approval number 201811050. | Location: Materials and Methods - Study subjects and animal studies |  |
| Studies involving experimental animals: State details of authority granting ethics approval (IRB or equivalent committee(s), provide reference number for approval.  Mouse studies: IACUC approval 72713 for University of Chicago, IACUC approval 20190101 for Washington University, St. Louis  NHP studies: Esaulova, E. et.al., (2021). The immune landscape in tuberculosis reveals populations linked to disease and latency. *Cell Host Microbe*, *29*(2), 165-178 e168. <https://doi.org/10.1016/j.chom.2020.11.013> | Location: Materials and Methods - Study subjects and animal studies |  |
| Studies involving specimen and field samples: State if relevant permits obtained, provide details of authority approving study; if none were required, explain why.  For MC staining on NHP samples, the IACUC protocol was under Dr. Deepak Kaushal’s lab and was published in Esaulova, E. et.al. (2021). The immune landscape in tuberculosis reveals populations linked to disease and latency. *Cell Host Microbe*, *29*(2), 165-178 e168. <https://doi.org/10.1016/j.chom.2020.11.013> | Location: Materials and Methods - Study subjects and animal studies |  |
|  |  |  |
| **Dual Use Research of Concern (DURC)** | **Indicate where provided: section/submission form** | **N/A** |
| If study is subject to dual use research of concern regulations, state the authority granting approval and reference number for the regulatory approval. |  | N/A |

**Analysis:**

| **Attrition** | **Indicate where provided: section/figure legend** | **N/A** |
| --- | --- | --- |
| Describe whether exclusion criteria were pre-established. Report if sample or data points were omitted from analysis. If yes, report if this was due to attrition or intentional exclusion and provide justification.  No pre- established exclusion criteria. All data points were included except those identified as outliers using Grubb’s outlier test | Location: Figure legends |  |
|  |  |  |
| **Statistics** | **Indicate where provided: section/figure legend** | **N/A** |
| Describe statistical tests used and justify choice of tests.  All data were analyzed using the indicated methodology in each figure legend. Two-sided unpaired t-test was performed for comparing the significance between 2 groups, one-way ANOVA Tukey’s test, and Sidak’s multiple comparison test were performed for more than 2 groups using GraphPad Prism 5 and 10, respectively | Location: Materials and Methods - Data analysis and statistics, and Figure legends |  |
|  |  |  |
| **Data availability** | **Indicate where provided: section/submission form** | **N/A** |
| For newly created and reused datasets, the manuscript includes a data availability statement that provides details for access (or notes restrictions on access).  No new sequencing datasets generated. All scRNA-seq analyses performed on publicly available datasets with accession numbers provided mentioned in Figure 2A and J. We have also included that in the data availability statement at the end of the manuscript. | Location: Figure legends |  |
| When newly created datasets are publicly available, provide accession number in repository OR DOI and licensing details where available. |  | N/A |
| If reused data is publicly available provide accession number in repository OR DOI, OR URL, OR citation.  Accession numbers: GSE149758 and GSE200151 | Location: Material and Methods- Single cell data reanalysis |  |
|  |  |  |
| **Code availability** | **Indicate where provided: section/figure legend** | **N/A** |
| For any computer code/software/mathematical algorithms essential for replicating the main findings of the study, whether newly generated or re-used, the manuscript includes a data availability statement that provides details for access or notes restrictions. |  | N/A |
| Where newly generated code is publicly available, provide accession number in repository, OR DOI OR URL and licensing details where available. State any restrictions on code availability or accessibility. |  | N/A |
| If reused code is publicly available provide accession number in repository OR DOI OR URL, OR citation. |  | N/A |

**Reporting:**

The MDAR framework recommends adoption of discipline-specific guidelines, established and endorsed through community initiatives.

| **Adherence to community standards** | **Indicate where provided: section/figure legend** | **N/A** |
| --- | --- | --- |
| State if relevant guidelines (e.g., ICMJE, MIBBI, ARRIVE, STRANGE) have been followed, and whether a checklist (e.g., CONSORT, PRISMA, ARRIVE) is provided with the manuscript. |  | N/A |

* We provide the following guidance regarding transparent reporting and statistics; we also refer authors to [Ten common statistical mistakes to watch out for when writing or reviewing a manuscript](https://doi.org/10.7554/eLife.48175).

**Sample-size estimation**

- You should state whether an appropriate sample size was computed when the study was being designed
- You should state the statistical method of sample size computation and any required assumptions
- If no explicit power analysis was used, you should describe how you decided what sample (replicate) size (number) to use

**Replicates**

- You should report how often each experiment was performed
- You should include a definition of biological versus technical replication
- The data obtained should be provided and sufficient information should be provided to indicate the number of independent biological and/or technical replicates
- If you encountered any outliers, you should describe how these were handled
- Criteria for exclusion/inclusion of data should be clearly stated
- High-throughput sequence data should be uploaded before submission, with a private link for reviewers provided (these are available from both GEO and ArrayExpress)

**Statistical reporting**

- Statistical analysis methods should be described and justified
- Raw data should be presented in figures whenever informative to do so (typically when N per group is less than 10)
- For each experiment, you should identify the statistical tests used, exact values of N, definitions of center, methods of multiple test correction, and dispersion and precision measures (e.g., mean, median, SD, SEM, confidence intervals; and, for the major substantive results, a measure of effect size (e.g., Pearson's r, Cohen's d)
- Report exact p-values wherever possible alongside the summary statistics and 95% confidence intervals. These should be reported for all key questions and not only when the p-value is less than 0.05.

**Group allocation**

- Indicate how samples were allocated into experimental groups (in the case of clinical studies, please specify allocation to treatment method); if randomization was used, please also state if restricted randomization was applied
- Indicate if masking was used during group allocation, data collection and/or data analysis
